# Myeloid-specific genetic ablation of ATP-binding cassette transporter ABCA1 is protective against cancer

**DOI:** 10.18632/oncotarget.18666

**Published:** 2017-06-27

**Authors:** Maryam Zamanian-Daryoush, Daniel J. Lindner, Joseph A. DiDonato, Matthew Wagner, Jennifer Buffa, Patricia Rayman, John S. Parks, Marit Westerterp, Alan R. Tall, Stanley L. Hazen

**Affiliations:** ^1^ Department of Cellular & Molecular Medicine, Cleveland Clinic, Cleveland, OH 44195, USA; ^2^ Taussig Cancer Institute, Cleveland Clinic, Cleveland, OH 44195, USA; ^3^ Department of Immunology, Cleveland Clinic, Cleveland, OH 44195, USA; ^4^ Department of Internal Medicine, Section on Molecular Medicine, Wake Forest School of Medicine, Winston-Salem, NC 27157, USA; ^5^ Department of Medicine, Columbia University, College of Physicians and Surgeons 8-401, New York, NY 10032, USA; ^6^ Department of Cardiovascular Medicine, Cleveland Clinic, Cleveland, OH 44195, USA

**Keywords:** cancer, apolipoprotein, myeloid cell, cholesterol, ABC transporter

## Abstract

Increased circulating levels of apolipoprotein A-I (apoA-I), the major protein of high-density lipoprotein (HDL), by genetic manipulation or infusion, protects against melanoma growth and metastasis. Herein, we explored potential roles in melanoma tumorigenesis for host scavenger receptor class B, type 1 (SR-B1), and ATP-binding cassette transporters A1 (ABCA1) and G1 (ABCG1), all mediators of apoA-I and HDL sterol and lipid transport function. In a syngeneic murine melanoma tumor model, B16F10, mice with global deletion of SR-B1 expression exhibited increased plasma HDL cholesterol (HDLc) levels and decreased tumor volume, indicating host SR-B1 does not directly contribute to HDL-associated anti-tumor activity. In mice with myeloid-specific loss of ABCA1 (*Abca1^−M/−M^*; A1^−M/−M^), tumor growth was inhibited by ∼4.8-fold relative to wild type (WT) animals. *Abcg1^−M/−M^* (G1^−M/−M^) animals were also protected by 2.5-fold relative to WT, with no further inhibition of tumor growth in *Abca1*/*Abcg1* myeloid-specific double knockout animals (DKO). Analyses of tumor-infiltrating immune cells revealed a correlation between tumor protection and decreased presence of the immune suppressive myeloid-derived suppressor cell (MDSC) subsets, Ly-6G^+^Ly-6C^Lo^ and Ly-6G^neg^Ly-6C^Hi^ cells. The growth of the syngeneic MB49 murine bladder cancer cells was also inhibited in A1^−M/−M^ mice. Collectively, our studies provide further evidence for an immune modulatory role for cholesterol homeostasis pathways in cancer.

## INTRODUCTION

Previously, we reported an anti-tumorigenic activity for apoA-I and HDL against melanoma [1]. Mice genetically manipulated to express human apoA-I (apoA-I Tg) were protected from tumor growth and metastases, whereas apoA-I-deficient (apoA-I KO) animals exhibited increased susceptibility to tumor proliferation [1]. Therapeutic delivery of human apoA-I into mice demonstrated anti-tumor activity in both established syngeneic mouse and xenograft human melanoma tumors. Both innate and adaptive immune arms were required for complete apoA-I anti-tumor activity, which was enabled in part through modulation of tumor infiltrating immune cells [1].

The athero-protective activities of HDL, namely lipid efflux and anti-inflammatory effects, are in major part thought to be mediated by the three known HDL or apoA-I receptors: ATP-binding cassette (ABC) transporters A1 (ABCA1) and G1 (ABCG1), and the scavenger receptor class B, type 1 (SR-B1) [2–4]. ABCA1 effluxes excess cholesterol and phospholipid to lipid-poor apoA-I, the predominant protein in HDL [5, 6], whereas ABCG1 mediates cholesterol and lipid transport to an already assembled HDL particle [7–9]. Efflux activity of ABCA1 initiates HDL assembly and defects in ABCA1-mediated cholesterol efflux lead to nearly non-detectable plasma HDL cholesterol (HDLc) levels in the mouse [10, 11] or Tangier disease in humans [12–15]. ABCA1 and ABCG1 are known to regulate several biological functions of hematopoietic cells. For example, mice deficient in ABCA1 and ABCG1 display hyper-proliferation of hematopoietic stem and progenitor cells (HSPCs), with specific expansion of the myeloid and granulocyte macrophage progenitors [2]. This expansion leads to increased mobilization and activation of myeloid cells, resulting in monocytosis and neutrophilia. Genetic knockdown of ABCA1 and ABCG1 also led to increased surface expression of Toll-like receptor 4 (TLR4) and heightened inflammatory gene expression [2, 16]. These observed effects were correlated with cholesterol accumulation in the transporter-deficient cells. Significantly, transplantation of bone marrow cells with genetic knockout of *Abca1* and *Abcg1* into mouse models prone to developing atherosclerosis resulted in accelerated atherosclerosis, underscoring an athero-protective role for these transporters in myeloid cells [2–4]. ABCA1 has been shown to possess anti-inflammatory activity, presumably via its cholesterol effluxing capacity to apoA-I [17]. ABCG1 similarly has anti-inflammatory activity, and is even more potent than ABCA1 in modulating cholesterol levels in the plasma membrane [16]. SR-B1, encoded by *Scarb1*, is the primary receptor for selective uptake of cholesteryl ester from HDL particles, and has also been reported to have an anti-inflammatory function in macrophages by suppressing Toll-like receptor-4 (TLR4) and nuclear factor-κB (NF-κB) signaling pathways [18].

In cancer cells, pharmacological and genetic evidence suggest a tumor-promoting role for cholesterol [19], and a tumor-inhibitory role for cholesterol efflux pathways [20]. For example, in prostate cancer cells inhibition of ABCA1, which effluxes cholesterol, resulted in tumor progression [19]. Conversely, elimination of SR-B1 in breast cancer cells, which overexpress this receptor, lead to inhibition in tumor growth, suggestive of a tumor permissive role for SR-B1 in cancer cells [21].

In contrast to their role in some cancer cells, cholesterol efflux transporters appear to promote a tumor-supportive function in immune cells. Tumor-mediated immune suppression is orchestrated by a number of infiltrating immune cell types including tumor associated macrophages (TAMs) and myeloid derived suppressor cells (MDSCs). TAMs and MDSCs both help promote tumor angiogenesis and metastasis, as well as tumor immune evasion. MDSCs are functionally and morphologically heterogeneous immature cell populations that fail to differentiate into mature dendritic cells, granulocytes, or macrophages, and are reported to inhibit the function of T cells, natural killer (NK) cells and other immune cells by different mechanisms [22]. Two main subsets of MDSCs have been defined: polymorphonuclear – MDSCs (Ly-6G^+^ Ly-6C^Lo^), which are granulocytic in appearance; and monocytic MDSCs (Ly-6G^neg^Ly-6C^Hi^) [23]. The anti-tumor function of apoA-I and HDL in the murine B16F10 melanoma tumor model is associated with changes in MDSCs and TAMs; however, the receptors involved and their impact on host immune surveillance remain unclear [1].

In a recent study, global and myeloid deficiency of ABCG1 was shown to be associated with reduced melanoma and bladder tumor growth in mice fed a “western”-type diet [24]. However, the functional role for alternative cholesterol efflux transporters, ABCA1 and SR-B1, in tumor growth and host immune surveillance, has not yet been reported. In this study we investigated the role of host cholesterol transporters ABCA1, ABCG1, and the HDL receptor SR-B1, on melanoma and bladder tumor growth. We report here that global deletion of SR-B1 protects mice against B16F10 melanoma tumor growth and myeloid-specific deletion of either ABCA1 or ABCG1, or the combined myeloid deletion of both ABCA1 and ABCG1 in somatic cells, protects mice against B16F10 melanoma tumor growth, with comparable results for ABCA1 in a syngeneic MB49 murine bladder cancer model. Our studies newly identify a potential melanoma tumor-permissive role for SR-B1 and myeloid ABCA1 in non-tumor somatic cells in tumor bearing hosts.

## RESULTS

### Global deletion of scavenger receptor class B, type 1 is protective against melanoma

**Figure 1:**
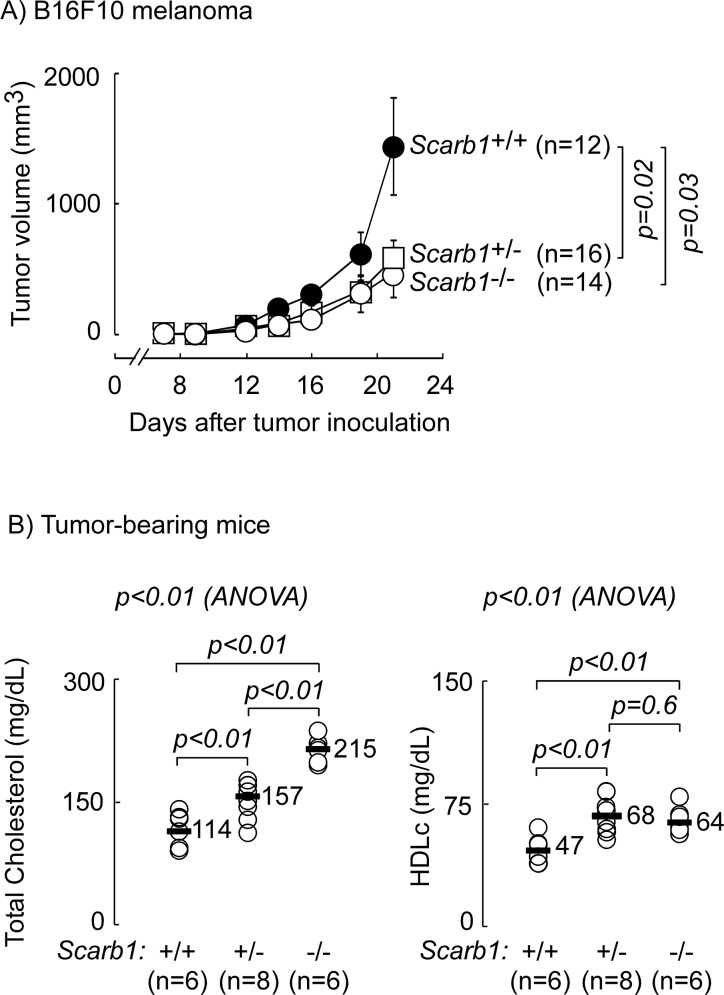
Global deletion of scavenger receptor B1 (SR-B1) is protective against melanoma C57BL/6 animals wild type (*Scarb1*^+/+^), hetero- (*Scarb1*^+/−^) or homozygously (*Scarb1*^−/−^) deleted at the locus for scavenger receptor type B, class 1 gene (*Scarb1*) were inoculated subcutaneously (10^5^ cells/flank) with B16F10 melanoma cells on day 0. **(A)** Tumor development was monitored by caliper measurements and tumor volume was calculated as described in Materials and Methods. n = number of inoculation sites, 2 sites/animal. Results shown are the mean ± SEM of a single experiment and are representative of repeat experiments. **(B)** Tumor bearing animals were sacrificed 21-days post tumor inoculation and plasma lipid distribution was determined as described in Materials and Methods. The median for each group is indicated. *p* values between two groups were determined by Student's *t*-test. Analysis of variance (ANOVA) across all three groups is shown above each graph.

To examine the impact of somatic cell SR-B1 on melanoma tumor growth, wild-type C57BL/6J mice (*Scarb1^+/+^*), or mice either heterozygous (*Scarb1*^+/−^) or homozygous (*Scarb1^−/−^*) for deletion of *Scarb1*, were inoculated subcutaneously with B16F10 melanoma cells (10^5^ cells/flank) and tumor growth was monitored over time by caliper measurements. If SR-B1 plays a role in the anti-tumor effect observed with apoA-I or HDL (1), we would anticipate enhanced tumor growth in the absence of SR-B1. In contrast, however, tumor growth in *Scarb1*^+/−^ mice was significantly (p<0.05) inhibited by approximately 3-fold relative to WT mice, with no significant further reduction in growth of tumor observed in *Scarb1^−/−^* mice (Figure 1A). Consistent with previous observations [25], significant increases in plasma total cholesterol (analysis of variance (ANOVA) between the groups p<0.01) were observed in mice with genetic deletion of *Scarb1* relative to WT mice (Figure 1B; 38% increase, WT vs *Scarb1*^+/−^, and 89% increase, WT vs *Scarb1^−/−^*). Further, lipoprotein subfractionation revealed a 44% increase in HDLc in *Scarb1*^+/−^ relative to WT, but no further increase in HDLc in *Scarb1^−/−^* (Figure 1B right panel). Interestingly, we observed a noticeable increase in LDLc in SR-B1 null mice (42±16, 73±13, and 128±7 mg/dL LDLc in WT, *Scarb1*^+/−^, and *Scarb1^−/−^*, respectively, data not shown). This suggests that in our tumor-bearing animals the increased total plasma cholesterol in SR-B1 null mice relative to *Scarb1*^+/−^ (Figure 1B), may have primarily partitioned to LDL instead of HDL particles. The observation in the present study that changes in HDLc are inversely correlated with tumor volume is consistent with our previous finding that higher HDL levels are associated with enhanced protection against melanoma [1]. Collectively, these results suggest that the anti-tumor activity of elevated HDL levels are independent of host SR-B1 and that loss of this receptor in host somatic cells hinders syngeneic transplanted melanoma tumor development.

### Mice with myeloid-specific deletion of ABCA1 are protected against melanoma and bladder cancer while additive deletion of ABCG1 does not provide further tumor inhibition

**Figure 2:**
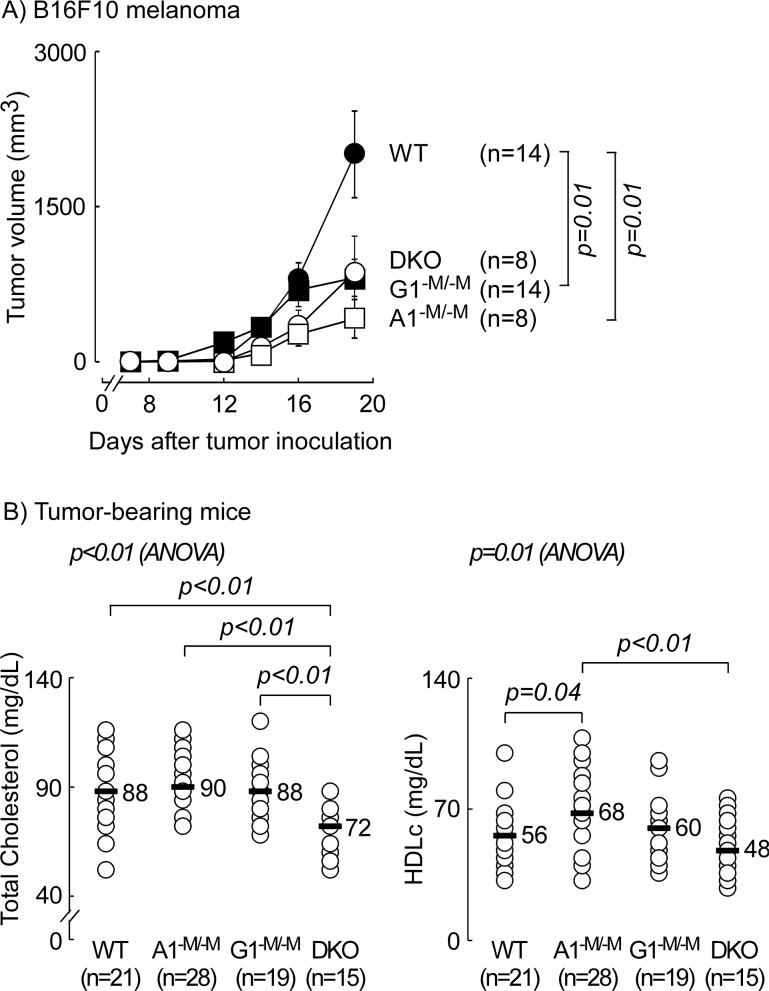
Mice with myeloid-specific deletion of ATP-binding cassette transporter ABCA1 are protected against melanoma- additive deletion in ABCG1 did not provide further protection C57BL/6 mice with wild type (WT; *LysM-Cre Abca1*^+/+^*Abcg1*^+/+^), myeloid deletion of *Abca1* (A1^−M/−M^), *Abcg1* (G1^−M/−M^), or *Abca1* and *Abcg1* (DKO) were inoculated with B16F10 tumor cells (10^5^ cells/flank) on day 0. **(A)** Tumor development was monitored by caliper measurements and tumor volume calculated as described in Materials and Methods. n = number of inoculation sites, 2 sites/animal. Results shown are the mean ± SEM. **(B)** Tumor bearing animals (B16F10 and MB49) were analyzed for plasma lipid distribution as described in Materials and Methods. The median for each group is indicated. *p* values between two groups were determined by Student's *t*-test. Analysis of variance (ANOVA) across all four groups is shown above each graph.

We previously reported that myeloid cells constitute a major immune cell infiltrate in B16F10 melanoma and that TAMs from apoA-I transgenic mice, which were protected from tumor development, had an M1- pro-inflammatory-like phenotype relative to TAMs from apoA-I knock-out mice [1]. Since SR-B1 does not appear to be involved in mediating this protective effect (Figure 1A), we hypothesized that myeloid cells deficient in either ABCA1, ABCG1 or both, which in multiple studies have previously been noted to have an inflammatory gene signature [2, 16, 26–28], might be protective in a B16F10 melanoma tumor model. To examine this possibility we used mice with myeloid-specific deletion of *Abca1* (A1^−M/−M^ [28]), *Abcg1* (G1^−M/−M^*),* or both *Abca1* and *Abcg1* (double knock-out (DKO) [2]). These animals express the Cre recombinase (*LysM*-*Cre*) in myeloid cells only, thus deleting loxP-floxed *Abca1* and *Abcg1* in a tissue-specific manner. In this study, the WT control mice also express the Cre recombinase in myeloid cells (*LysM*-*Cre*) but lack loxP sites in *Abca1* and *Abcg1* genes. Animals were inoculated with B16F10 melanoma cells (10^5^ tumor cells/flank) and tumor development over time was quantified. Relative to WT mice, tumor growth was inhibited (on day 19 post inoculation) by 4.8-, 2.5-, and 2.3-fold in A1^−M/−M^, G1^−M/−M^, and DKO, respectively (Figure 2A). Importantly, loss of myeloid *Abca1* alone was sufficient to significantly inhibit tumor development (p=0.01), and additional deletion of *Abcg1* in the same immune cells did not lead to enhanced suppression of tumor growth (Figure 2A). Furthermore, the anti-tumor effects observed were noted in relatively young (3-4 months) animals on regular chow diet and did not require either aged mice (6-7 months old) or a high fat “western”-type diet, as was recently reported as a requirement for tumor suppressive phenotype in *Abcg1^−/−^* mice [24]. ANOVA analysis of plasma lipid profiles across the groups showed significant differences in total cholesterol and HDLc (p<0.01). Moreover, while A1^−M/−M^ or G1^−M/−M^ mice showed no difference in total cholesterol level relative to WT, the myeloid specific DKO animals exhibited a modest (18-20%) but statistically significant decrease in plasma total cholesterol levels (p<0.01) relative to WT and each of the single receptor knock-out mice (Figure 2B, left panel). The reduction in circulating cholesterol in the DKO mice did not translate into a significant difference in HDLc between WT and DKO (Figure 2B, right panel). Hepatic (not myeloid) ABCA1 has been reported to be the major determinant of plasma HDLc levels [29]. Therefore, the modest but statistically significant increase in HDLc in the A1^−M/−M^ group of mice was unexpected and may contribute to the tumor protection observed in this group, though a similar increase in HDLc was not observed in G1^−M/−M^ or DKO (Figure 2B, right panel).

**Figure 3:**
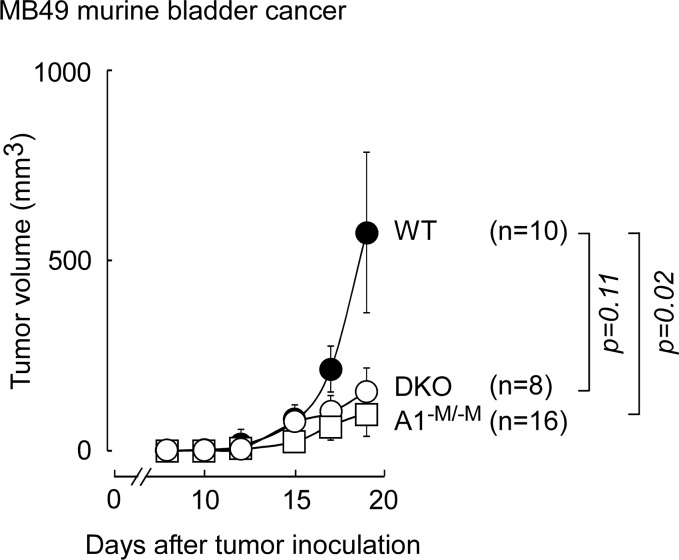
Mice with myeloid-specific deletion of ABCA1, are protected against MB49 bladder cancer on normal chow diet C57BL/6 mice with wild type (WT; *LysM-Cre Abca1^+/+^Abcg1*^+/+^), myeloid deletion of *Abca1* (A1^−M/−M^) or *Abca1* and *Abcg1* (DKO) were inoculated with 10^5^ MB49 cells/flank on day 0. Tumor development was monitored by caliper measurements and tumor volume calculated as described in Materials and Methods. n = number of inoculation sites, 2 sites/animal. Results shown are the mean tumor volume ± SEM. *p* values between two groups were determined by Student's *t*-test.

We next examined whether the tumor inhibition observed in animals with myeloid ablation of ABCA1 was specific to B16F10 melanoma. Animals (6-7 months old) were inoculated subcutaneously (10^5^ cells/flank) with an alternative syngeneic tumor model, MB49 bladder carcinoma cells, and tumor progression over time was subsequently followed by caliper measurements. After 19-days there was a 5.7-fold inhibition in tumor volume in the A1^−M/−M^ animals relative to WT controls (p=0.02), and a similar trend toward inhibition in DKO animals, although the numbers did not reach statistical significance (Figure 3). In contrast to B16F10 melanoma, we did not observe tumor protection in younger A1^−M/−M^ mice (data not shown). These results suggest that the therapeutic targeting of myeloid ABCA1 may be effective against a wide spectrum of cancers.

### Myeloid-specific deletion of ABCA1 and ABCG1 inhibits tumor bed accumulation of MDSCs

**Figure 4:**
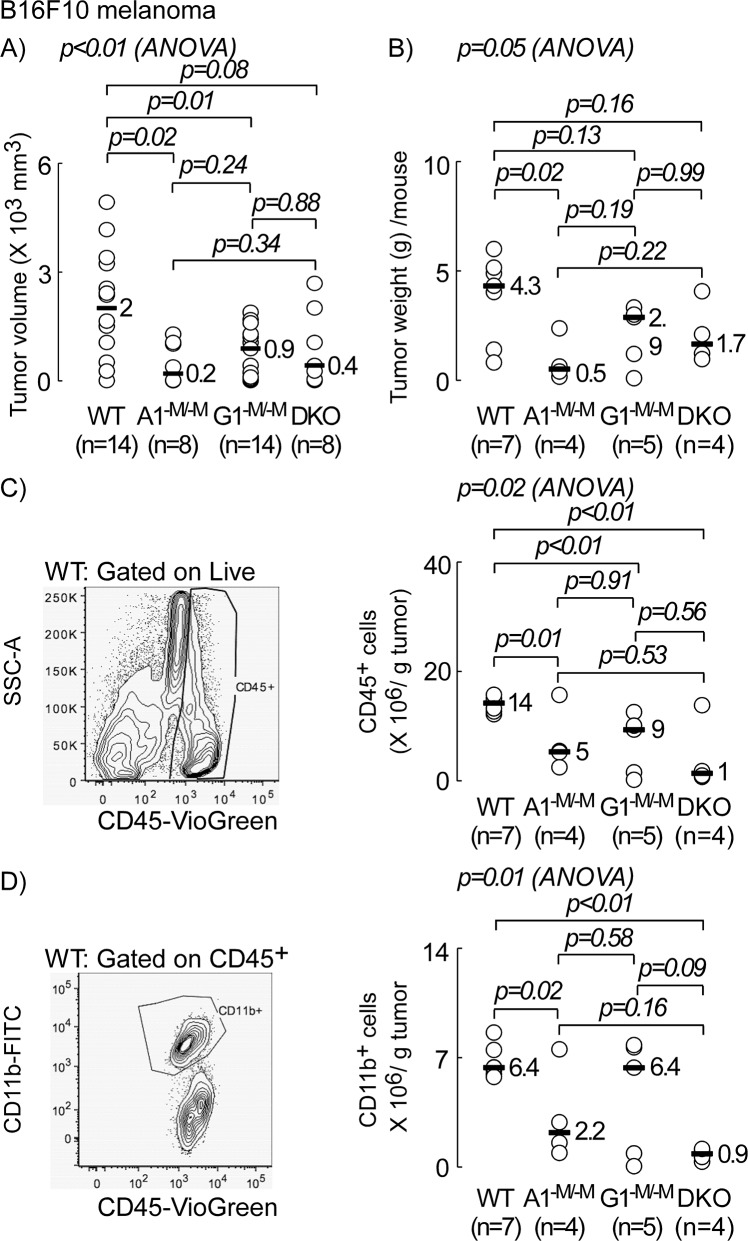
Myeloid-specific deletion of ABCA1 and ABCG1 transporters inhibits accumulation of leukocytes in tumor bed C57BL/6 mice with wild type (WT; *LysM-Cre Abca1*^+/+^*Abcg1*^+/+^), myeloid deletion of *Abca1* (A1^−M/−M^), *Abcg1* (G1^−M-M^) or *Abca1* and *Abcg1* (DKO) were inoculated with B16F10 tumor cells (10^5^ cells/flank) and sacrificed 19-days later. **(A)** Individual tumor volumes (2 per animal) as determined by caliper measurement (see Materials and Methods) on day of sacrifice, day 19. **(B)** Pooled tumor weight per animal which were processed for FACs analyses. **(C)** Number of tumor infiltrating leukocytes (CD45^+^) per gram tumor tissue. Cells were gated on live. Resected tumors (two per animal) from individual mice (n=7, WT; n= 4, A1^−M/−M^ and DKO, and n=5, G1^−M/−M^) were digested to obtain single cells for surface antigen staining and analysis by flow cytometry, as described in Materials and Methods. **(D)** Number of tumor infiltrating CD11b^+^ cells per gram tumor tissue. Cells were gated on live and CD45^+^. Representative FACs images to the left are WT Control and are gated on live **(C)**, and live/CD45^+^ **(D)**. *p* values between two groups were determined by Student's *t*-test. Analysis of variance (ANOVA) across all four groups is shown above each graph.

**Figure 5:**
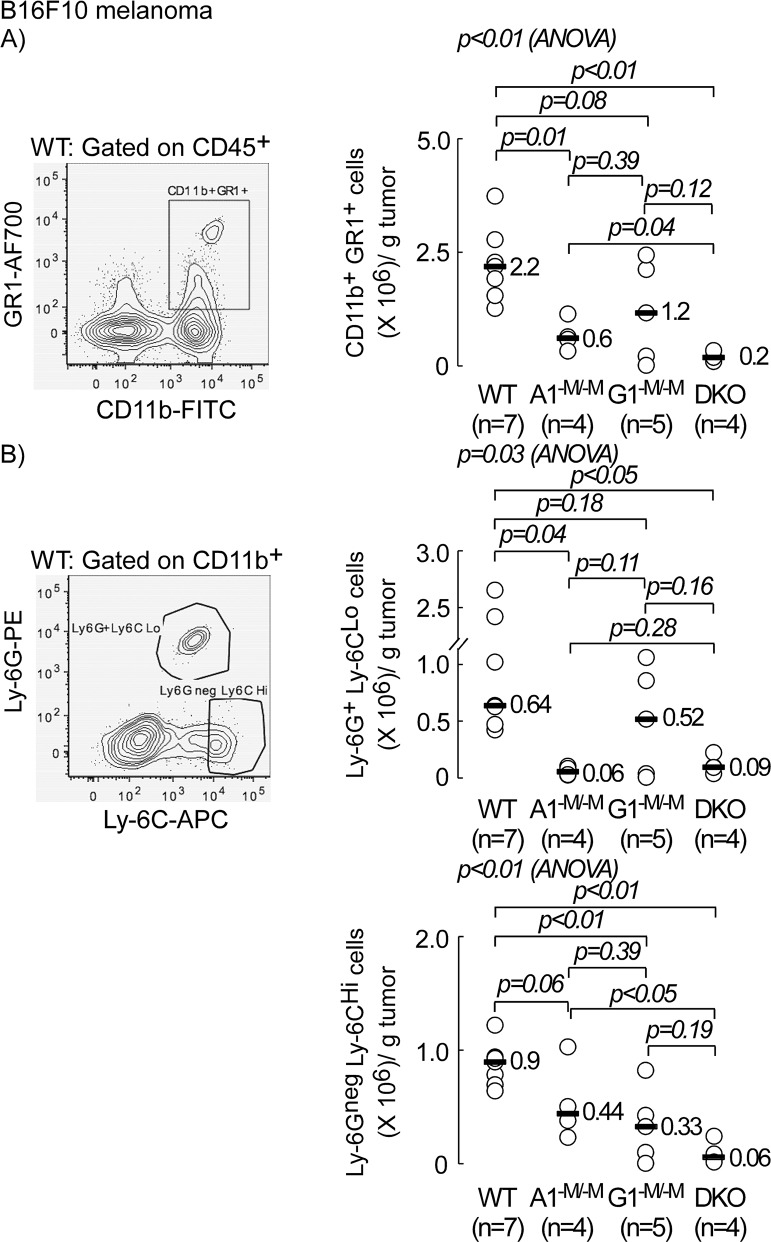
Myeloid-specific deletion of ABCA1 and ABCG1 transporters inhibits accumulation of myeloid derived suppressor cells (MDSCs) in tumor bed C57BL/6 mice with wild type (WT; *LysM-Cre Abca1*^+/+^*Abcg1*^+/+^), myeloid deletion of *Abca1* (A1^−M/−M^), *Abcg1* (G1^−M-M^) or *Abca1* and *Abcg1* (DKO) were inoculated with B16F10 tumor cells (10^5^ cells/flank) and sacrificed 19-days later. Resected tumors (two per animal) from individual mice (n=7, WT; n= 4, A1^−M/−M^ and DKO, and n=5, G1^−M/−M^) were digested to obtain single cells for surface antigen staining and analysis by flow cytometry, as described in Materials and Methods. **(A)** Number of CD11b^+^GR1^+^ (MDSCs) per gram tumor tissue. **(B)** Number of Ly-6G^+^Ly-6C^Lo^ (granulocytic MDSCs) and Ly-6G^neg^Ly-6C^Hi^ (monocytic MDSCs) per gram tumor. Representative FACs images to the left are WT Control and are gated on live/CD45^+^ (A), and on live/CD45^+^/CD11b^+^ (B). *p* values between two groups were determined by Student's *t*-test. Analysis of variance (ANOVA) across all four groups is shown above each graph.

Tumor proliferation is largely influenced by infiltrating immune cells, which are recruited and subverted by the tumor microenvironment to promote tumor growth and metastasis. Since myeloid cells constitute the majority of infiltrating immune cells in B16F10 melanoma [1], we sought to examine if myeloid-specific transporter deletions influenced the composition of infiltrating leukocytes within the tumor bed. After inoculation of B16F10 cells, tumors were excised (day 19) and processed for FACs analyses, as described in Materials and Methods. Host immune cells were differentiated from tumor cells by surface staining for the hematopoietic leukocyte common antigen, CD45 [30]. In Figure 4A and 4B, tumor volume and weight are shown for each group of mice. There was a 10-, 2.3-, and 4.7-fold inhibition in median tumor volume on the day of sacrifice in A1^−M/−M^, G1^−M/−M^ and myeloid-specific DKO mice, respectively relative to control WT mice, with only A1^−M/−M^ and G1^−M/−M^, but not myeloid-specific DKO mice, showing significant differences (p=0.02, p=0.01, and p=0.08, respectively). The remaining panels in Figures 4 and 5 include contour diagrams to the left to illustrate gating used during flow cytometry, and the panels to the right represent scatter plots quantifying the number of tumor-infiltrating cells for the indicated surface antigens per gram tumor tissue in all four groups. The number of infiltrating CD45^+^ cells/g tumor was reduced by 2.8-, 1.6-, and 14-fold in A1^−M/−M^, G1^−M/−M^ and DKO animals relative to WT (p=0.01, p<0.01, and p<0.01, respectively, Figure 4C). Myeloid cells (CD11b^+^) constituted nearly 50% or greater of these tumor infiltrating leukocytes (Figure 4D). Although there was a similar trend (decrease in receptor knock-out mice relative to WT) in levels of CD11b^+^F4/80^+^ macrophages (ANOVA p=0.15), the decrease was only statistically significant between WT and DKO (6-fold decrease, p<0.01, data not shown).

**Figure 6:**
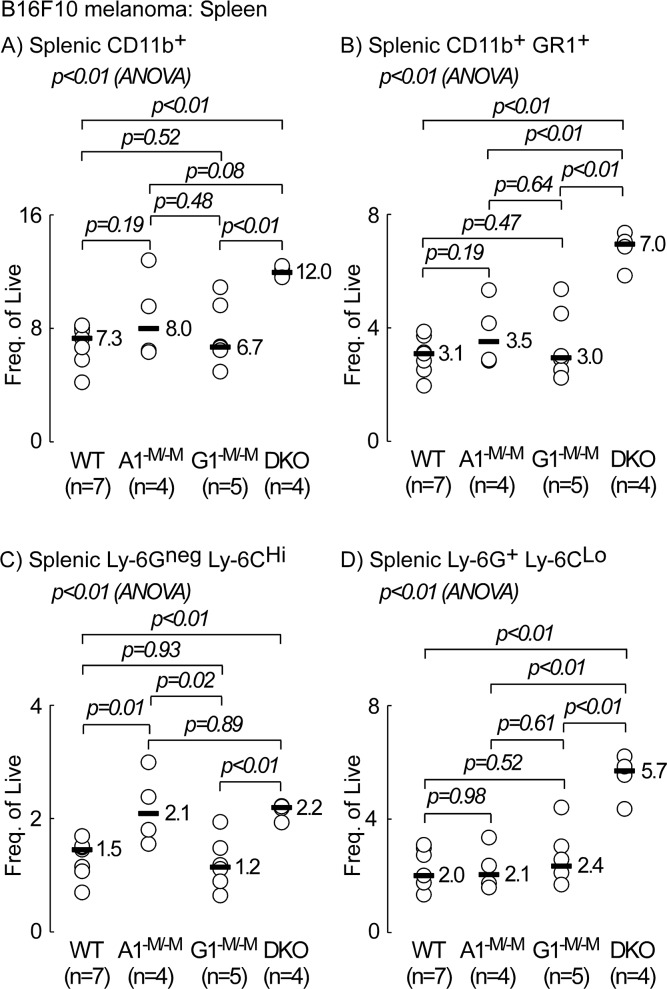
Animals with myeloid-specific deletion of both ABCA1 and ABCG1 transporters (DKO) exhibit higher frequencies of splenic CD11b^+^, CD11b^+^GR1^+^, monocytic and granulocytic MDSCs C57BL/6 mice with wild type (WT; *LysM-Cre Abca1*^+/+^*Abcg1*^+/+^), myeloid deletion of *Abca1* (A1^−M/−M^), *Abcg1* (G1^−M/−M^), and myeloid deletion of *Abca1* and *Abcg1* (DKO) were inoculated with B16F10 tumor cells (10^5^ cells/flank) and sacrificed 19-days later. Individual spleens were processed for FACs analysis as described in Materials and Methods. Cells were gated on live for analysis. **(A)** Frequency of splenic CD11b^+^ cells. **(B)** Frequency of CD11b^+^GR1^+^. **(C)** Frequency of Ly-6G^neg^Ly-6C^Hi^. **(D)** Frequency of Ly-6G^+^Ly-6C^Lo^. *p* values between two groups were determined by Student's *t*-test. Analysis of variance (ANOVA) across all four groups is shown above each graph.

In mice, MDSCs are broadly characterized as CD11b^+^GR1^+^, where the myeloid differentiation antigen GR1 consists of two epitopes, Ly-6G and Ly-6C [23]. There were fewer MDSCs in tumors from all receptor knock out groups relative to WT controls (3.7-, 1.8-, and 11-fold decrease in A1^−M/−M^, G1^−M/−M^, and DKO, respectively; p=0.01, p=0.08 and p<0.01, respectively, Figure 5A). Importantly, myeloid ablation of ABCA1 resulted in an almost 11-fold decrease in granulocytic MDSCs, Ly-6G^+^Ly-6C^Lo^ (median= 0.64 and 0.06 × 10^6^ cells/gram tumor tissue in WT and A1^−M/−M^, respectively, p=0.04, Figure 5B, top plot). There was no statistically significant difference between G1^−M/−M^ and WT (p=0.18, Figure 5B, top plot) but a significant 7-fold decrease in Ly-6G^+^Ly-6C^Lo^ cells in DKO (median= 0.64 and 0.09 × 10^6^ cells/gram tumor tissue in WT and DKO, respectively, p<0.05, Figure 5B, top plot). In contrast, myeloid ablation of ABCG1 resulted in a statistically significant decrease (2.7-fold) in numbers of monocytic MDSCs, Ly-6G^neg^Ly-6C^Hi^ (median=0.9 and 0.33 × 10^6^ cells/gram tumor tissue in WT and G1^−M/−M^, respectively, p<0.01, Figure 5B, lower plot). There was even further decrease (15-fold) in the monocytic MDSC subset in DKO (median=0.9 and 0.06 × 10^6^ cells/gram tumor tissue in WT and DKO, respectively, p<0.01, Figure 5B, lower plot). There was no statistically significant difference in monocytic MDSCs between A1^−M/−M^ and WT (p=0.06, Figure 5B, lower plot). These results suggest that myeloid ABCA1 may be important in the accumulation of granulocytic MDSCs (Ly-6G^+^Ly-6C^Lo^) whereas myeloid ABCG1 may govern the frequency of monocytic MDSCs (Ly-6G^neg^Ly-6C^Hi^) in the tumor microenvironment. The lower numbers of MDSCs in animals with myeloid ablation of ABCA1 and ABCG1 was specific to the tumor compartment as these cells were not decreased in the spleen of receptor knock-out mice relative to WT animals (Figure 6B, 6C, and 6D). In contrast to the tumor (Figure 4D), the frequency of CD11b^+^ cells increased by 1.6-fold in spleens of DKO (Figure 6A, p<0.01). The frequency of Ly-6G^neg^ Ly-6C^Hi^ cells was increased modestly (∼1.5-fold) in the spleen of both A1^−M/−M^ and DKO animals relative to WT (Figure 6C, p=0.01 and p<0.01, respectively) and that of Ly-6G^+^ Ly-6C^Lo^ cells was increased only in DKO (2.8-fold relative to WT, Figure 6D, p<0.01).

Analysis of complete blood cell count (CBC) with differentials in naïve (non-tumor bearing) mice revealed no significant differences between WT, A1^−M/−M^ and DKO except for a mild but statistically significant elevation of monocytes in DKO vs A1^−M/−M^ (0.3 ± 0.04 vs 0.09 ± 0.03 X10^3^ cells/μl blood in DKO and A1^−M/−M^, respectively, p<0.01; data not shown). Tumor-bearing (B16F10 melanoma) mice similarly showed no major defects in CBC + differential, except for a statistically significant decrease in numbers of circulating monocytes and neutrophils in A1^−M/−M^ animals relative to WT, G1^−M/−M^ or DKO (Table 1). Further, a trend towards elevated neutrophil count in tumor bearing G1^−M/−M^ and DKO mice relative to WT and A1^−M/−M^ mice was observed (Table 1).

**Table 1:** Mice with myeloid specific deletion of ATP-binding cassette transporter ABCA1 exhibit reduced circulating monocytes and neutrophils

Cells/μl	WT (n=12)	A1^−M/−M^ (n=11)	G1^−M/−M^ (n=10)	DKO (n=6)	A1^−M/−M^ vs WT *p*-value	G1^−M/−M^ vs WT *p*-value	DKO vs WT *p*-value	A1^−M/−M^ vs G1^−M/−M^ *p*-value	A1^−M/−M^ vs DKO *p*-value	G1^−M/−M^ vs DKO *p*-value
WBC (10^3^)	3.93 ± 0.78	2.22 ± 0.31	3.35 ± 0.61	3.63 ± 0.49	0.063	0.575	0.798	0.106	0.023	0.757
Neut (10^3^)	1.03 ± 0.23	0.49 ± 0.1	1.38 ± 0.43	1.47 ± 0.45	0.046	0.460	0.338	0.050	0.014	0.891
Lymph (10^3^)	2.48 ± 0.54	1.57 ± 0.23	1.65 ± 0.21	1.88 ± 0.14	0.153	0.202	0.457	0.809	0.373	0.453
Mono (10^3^)	0.32 ± 0.09	0.08 ± 0.01	0.25 ± 0.05	0.17 ± 0.03	0.026	0.581	0.285	0.005	0.018	0.281
Eos (10^3^)	0.02 ± 0.00	0.02 ± 0.00	0.02 ± 0.00	0.04 ± 0.00	0.938	0.470	0.136	0.532	0.122	0.001
Baso (10^3^)	0.06 ± 0.02	0.05 ± 0.03	0.02 ± 0.01	0.04 ± 0.01	0.877	0.100	0.457	0.339	0.708	0.217
RBC (10^6^)	3.79 ± 0.53	3.96 ± 0.82	2.93 ± 0.55	5.41 ± 0.79	0.860	0.270	0.099	0.315	0.265	0.018
PLT (10^4^)	53.53 ± 7.40	50.90 ± 11.33	45.17 ± 8.76	69.45 ± 10.46	0.846	0.471	0.232	0.697	0.301	0.103
HGB (g/dL)	4.64 ± 0.91	5.97 ± 1.26	4.36 ± 0.99	7.92 ± 1.14	0.395	0.836	0.046	0.334	0.326	0.039
HCT (%)	19.46 ± 2.42	19.20 ± 3.89	16.37 ± 2.86	26.10 ± 3.36	0.955	0.417	0.131	0.572	0.258	0.050

Results from complete blood counts and differentials from tumor-bearing (B16F10 melanoma) mice on chow diet recovered at time of sacrifice by cardiac puncture, as described in Materials and Methods. Number of mice in each group is indicated (n). Results shown are the mean ± SEM. *p*-values were determined by Student's *t* –test for the indicated comparison.

## DISCUSSION

Recently, several reports have collectively provided evidence for an immune modulatory role for cholesterol homeostasis pathways in cancer. Notably, large-scale human epidemiological studies have noted an inverse association between circulating HDLc levels and cancer risk [31, 32]. Furthermore, reduced plasma level of apoA-I, the predominant protein in HDL, was identified as a biomarker in patients with early-stage ovarian cancer [33–38]. Animal studies have similarly demonstrated that elements of cholesterol homeostasis pathways impact tumor growth and development. For example, apoA-I/HDL, a lipoprotein involved in cholesterol transport in the circulation, was shown to have anti-tumor activity against both syngeneic and xenogeneic tumor models in mouse [1, 39]. The ATP binding cassette transporters ABCA1 and ABCG1 promote efflux of excess cholesterol and lipids from peripheral cells to circulating HDL for delivery to the liver and excretion into bile and feces in a physiological process known as reverse cholesterol transport [40, 41]. ABCG1 was recently shown to influence the anti-tumor function of immune cells. Both global and myeloid deletion of *Abcg1* led to increased tumor immunity in young tumor bearing animals when fed a “western”-type diet, but not a normal chow diet [24]. Interestingly, in aged animals (6-7 months old), the protection associated with *Abcg1* deficiency was observed in chow-fed animals [24].

In the present study, we extend these observations, and examine the potential role of two distinct apoA-I/HDL receptors in tumor biology. Global knockout of *Scarb1* (SR-B1) in 2-3 month old tumor bearing animals on chow diet led to inhibition of B16F10 tumor development compared to WT animals (Figure 1A) and increased levels of circulating HDLc (Figure 1B). Further, mice with myeloid-specific deletion of *Abca1* (A1^−M/−M^) were protected against tumor development in relatively young (3-4 months old) animals on normal chow diet (Figure 2A). G1^−M/−M^ mice were also protected under these experimental conditions, though not as robustly as A1^−M/−M^ (Figure 2A). Importantly, A1^−M/−M^ mice were also notable for being resistant to MB49 bladder cancer, suggesting that the protective mechanism triggered by loss of myeloid ABCA1 may be effective against a wide spectrum of tumors (Figure 3A).

We previously showed an innate and adaptive immune-modulatory role for apoA-I levels in melanoma tumor biology [1]. Our present study suggests that the tumor protective effect of increasing apoA-I/HDL levels likely did not function through SR-B1, the primary receptor for HDL particles and a key regulator of total cholesterol levels [42, 43]. Tumor growth was significantly inhibited in animals heterozygous or homozygous for deletion in SR-B1 (Figure 1A). Of interest, a role for SR-B1 in adaptive immunity has previously been suggested since *Scarb1^−/−^* mice were shown to have increased expression of the proinflammatory cytokines TNFα, and IL-6 and inducible nitric oxide synthetase in circulation, increased frequency of splenic macrophages and impaired lymphocyte homeostasis with activated T and B cells [44]. Additionally, a further link between HDL and macrophage immune function was suggested by the observation that SR-B1 can mediate bidirectional cholesterol transfer between macrophages and HDL [45, 46]. Of particular note, SR-B1-null mice were reported to have hyperinflammatory macrophages independent of altered cellular or membrane cholesterol content [47]. Whether the observed tumor immunity in our present study is associated with these previously reported phenotypic changes in immune cells of *Scarb1*-null mice is unknown.

In prior studies, the anti-neoplastic activity of apoA-I and HDL [1], and the tumor protection observed with *Abcg1* deficiency [24], were suggested to be mediated in part by the myeloid immune cell subset. In particular, macrophages were noted to switch to an M1-like tumor-fighting phenotype. Thus, TAMs recovered from tumor-bearing apoA-I transgenic mice had a pro-inflammatory M1-like gene signature and peritoneal macrophages from these animals displayed enhanced cytotoxicity towards tumor cells relative to macrophages recovered from tumor-bearing apoA-I-null mice [1]. Myeloid-specific loss of ABCA1 and or ABCG1 expression has been shown to impart a pro-inflammatory phenotype to these immune cells [2, 16, 24, 48, 49]. In the present study, we therefore hypothesized that mice with myeloid-specific *Abca1* deletion may exhibit resistance to tumor development. Our observations with chow-fed, tumor bearing mice are supportive of this hypothesis, and consistent with our previous observation that pro-inflammatory TAMS inhibit B16F10 tumor development [1].

The recently observed [24] anti-tumor phenotype with *Abcg1* deficiency was also noted to be accompanied by enhanced *Abca1* expression in TAMs, though the potential role of ABCA1 was not examined. In the present study, we show that myeloid loss of *Abca1* alone was sufficient to confer enhanced tumor immunity in both melanoma (Figure 2) and bladder cancer (Figure 3). We also saw no further inhibition in the rate of tumor development by the additional loss of *Abcg1* in myeloid cells (Figure 2A; compare DKO to A1^−M/−M^). Moreover, unlike the situation reported with ABCG1*-*myeloid deficiency where the tumor protective effect was observed under conditions of hypercholesterolemia [24], this does not appear to be a prerequisite to elicit tumor protection with ABCA1-myeloid deficiency.

Here, we identified a tumor protective function against B16F10 melanoma and MB49 bladder cancer in mice with myeloid deficiency in ABCA1 (A1^−M/−M^) or ABCA1 and ABCG1 (DKO). It is possible that this protection may be related to the previously reported proinflammatory anti-tumor phenotype (M1) of TAMS in these animals, a hypothesis that deserves further study [1, 24]. In the present studies, while we saw no statistically significant difference in the numbers of TAMs per gram tumor between different genotypes (data not shown), we did observe a lower frequency of MDSCs in general (CD11b^+^GR1^+^), as well as monocytic (Ly-6G^neg^Ly-6C^Hi^) and granulocytic MDSCs (Ly-6G^+^Ly-6C^Lo^) in these animals (Figure 5A and 5B, respectively). We acknowledge that the correlation between MDSC prevalence and tumor burden is merely associative at this point and further experiments are needed to prove a causal relationship. We speculate the decrease in myeloid infiltration may be related to the inflammatory microenvironment around the tumor [50] since prior studies have shown A1^−M/−M^ or DKO macrophages exhibit increased apoptosis and decreased migration into inflammatory settings [51]. It is also notable that Hedrick and colleagues similarly recently observed a lower frequency of macrophage infiltration into MB49 tumors in G1^−M/−M^ mice, as well as enhanced macrophage apoptosis in “western”-type diet fed *Abcg1^−/−^* MB49 tumor bearing mice [24]. On a chow diet, ABCA1 and ABCG1 protein expression was shown to be reduced by >95% in macrophages from DKO [2]. DKO monocytes are reported to show >80% reduction in ABCA1 and ABCG1, whereas in neutrophils there was a >50% reduction in ABCA1, but no significant reduction in ABCG1 mRNA [2]. This may explain the synergistic reduction in monocytic MDSCs (Ly-6G^neg^Ly-6C^Hi^) in tumors from DKO animals (Figure 5B, lower plot) but not that of granulocytic MDSCs (Ly-6G^+^Ly-6C^Lo^) (Figure 5B, top plot). Additional factors may be influencing MDSC numbers in tumors from DKO animals. For example, it has been shown that *Ldlr^−/−^* mice transplanted with DKO bone marrow displayed neutrophilia on chow diet and monocytosis in addition to enhanced neutrophilia on western-type diet [2]. In the present study however, tumor bearing myeloid DKO animals did not exhibit statistically significant increased circulating levels of monocytes or neutrophils (Table 1). This discrepancy may be due to differences in genetic background (*Ldlr^−/−^*), diet (Western vs chow), or tumor-driven effects in our study versus the previously published report [2]. Interestingly, we observed a significant increase in splenic CD11b^+^, CD11b^+^GR1^+^, Ly-6G^neg^Ly-6C^Hi^ and Ly-6G^+^Ly-6C^Lo^ cells in DKO (Figure 6), arguing against defective migration as the sole underlying cause for lower frequency of MDSCs in tumors from DKO mice. Increased apoptosis in receptor null myeloid cells [51, 52] is another potential contributor to the lower observed MDSC levels in receptor null animals. In contrast, A1^−M/−M^ tumor bearing animals had significantly reduced levels of blood monocytes and neutrophils (Table 1), a trend that was not reflected in the spleen (Figure 6). It is thus plausible that in A1^−M/−M^ animals MDSCs may not accumulate in the tumor bed in part because of lower levels of circulating monocytic immune cells.

Our present studies add to the growing support for the notion that loss of ABCA1 and ABCG1 in myeloid cells modulates tumorigenesis through impacting the immune system. They also have several limitations worth noting. The observation that animals with myeloid deficiency of ABCA1 (A1^−M/−M^) and or ABCG1 (G1^−M/−M^) transporters had smaller B16F10 tumors and reduced accumulation of tumor-infiltrating MDSCs is, at present, only associative. Further investigation into the role of MDSCs in mediating the observed influence of myeloid *Abca1* gene in tumorigenesis are needed to prove a specific role of this cell type in the observed effects on tumor growth. While the underlying mechanism of action by these cholesterol transporter proteins remain to be fully understood, it is of interest to note that previous reports have shown that modulating cholesterol levels at the plasma membrane, either biochemically or through the deletion of cholesterol transporters, can impact immune cell function and phenotype through changes in cellular signaling [2, 16, 24, 27, 28]. Thus, loss of ABCA1/ABCG1 in our experimental setting may have altered macrophage signaling, as previously shown in *Abca1^−/−^, Abcg1^−/−^* or DKO macrophages [16]. For example, heightened TLR signaling is associated with deletion of the cholesterol transporters [16], and TLR signaling has been shown to play a crucial role in initiation of innate immune responses against cancer and subsequent induction of adaptive immune responses [53]. The role of TLR signaling in the present studies remain to be explored. It is reported that TLR signaling in both tumor cells and host immune cells is complex, with both potential pro-tumorigenic advantages to tumor cells and anti-tumor immunogenic immune cell responses to endogenous tumor-generated TLR ligands [54–56]. In addition to changing the immune cell phenotype, altered signaling in receptor-null immune cells may also affect the migration of these cells and their ability to home in to the tumor. One can also speculate that reduced MDSC accumulation in tumors from receptor-null animals may thus in part be explained by defective ‘homing’, as well as enhanced apoptosis [24, 51]. In normal inflammatory settings, apoA-I/HDL suppresses inflammatory responses in macrophages [57–59]. In the tumor microenvironment, apoA-I/HDL appears to promote inflammation by inducing the accumulation of M1-like pro-inflammatory TAMs that have anti-tumor activity [1]. The molecular basis for this shift in TAM phenotype in tumor-bearing mice is under further investigation, but in essence it is similar to our present observations for the myeloid-deleted transporters and those reported by Hedrick and colleagues [24]. Thus, it appears that the loss of ABCA1/ABCG1 in myeloid cells “hard-wires” these immune cells to become pro-inflammatory.

In conclusion, our present study adds to an emerging theme that cholesterol metabolism and tumor immunity are closely linked. Multiple clinical studies have shown an inverse association between circulating HDLc or apoA-I levels and cancer risk [31–38]. Furthermore, studies with liver X receptor (LXR) antagonists resulting in immune cell cholesterol accumulation indicate increased dendritic cell infiltration into tumors with beneficial effects [60, 61]. Most recently, apoA-I and myeloid ABCG1 have been shown to influence the anti-tumor function of immune cells [1, 24]. In the present studies, inhibiting cholesterol efflux pathways globally with SR-B1, or more specifically in myeloid cells by alternative approaches, including targeted deletion of the transporters ABCA1 alone, or ABCA1 in combination with ABCG1, resulted in significant reduction in tumor growth, and accompanying reduction in frequency of the immunosuppressive and tumor-promoting MDSCs. Further studies exploring both the mechanisms linking myeloid cholesterol transport and immune protection against cancer, and the anti-tumor potential of therapies that target immune cell cholesterol content, seem warranted.

## MATERIALS AND METHODS

### Materials and general procedures

All chemicals were from Sigma Chemical (St. Louis, MO) and all solvents were HPLC grade unless otherwise indicated. All mouse studies were performed under protocols approved by the Institutional Animal Care and Use Committee at the Cleveland Clinic.

### Mice

Scavenger receptor class B, type 1 null (*Scarb1^−/−^*) mice used were back-crossed onto a C57BL/6J background for >>10 generations. *LysM*-*Cre Abca1^−M/−M^ Abcg1^+/+^* mice (referred to as A1^−M/−M^ in this study) were provided by Dr. John Parks (Wake Forest University). Drs. Marit Westerterp and Alan Tall (Columbia University) provided *LysM*-*Cre Abca1^−M/−M^Abcg1^−M/−M^* mice (referred to as DKO in this study). *LysM*-*Cre Abcg1^−M/−M^* (referred to as G1^−M/−M^ in this study) was bred out of DKO in our mouse facility by initially crossing the DKO with WT (*LysM*-*Cre Abca1*^+/+^*Abcg1*^+/+^) to obtain *LysM*-*Cre Abca1*^+/+^*Abcg1*^−*M*/+^ and then crossing these animals together to obtain homozygous floxed *Abcg1* (*LysM*-*Cre Abca1*^+/+^*Abcg1^−M/−M^*). Both myeloid-deleted ABC-transporter strains were backcrossed onto a C57BL/6J background for >>10 generations. C57BL/6J mice (referred to as wild type (WT)) were *LysM-Cre Abca1*^+/+^*Abcg1*^+/+^ and were provided by Dr. John Parks. All mice were bred at Cleveland Clinic's Biological Research Unit (BRU).

### Tumor cell lines

Mouse tumor cell line B16F10 melanoma was obtained from American Type Culture Collection (CRL-6475, ATTC, Bethesda, MD) and MB49 bladder cancer cells were a gift from Ernest Borden (Cleveland Clinic) and were established in 1979 following dimethylbenzanthracene treatment of C57BL/6J mice. Tumor cells were cultured in DMEM supplemented with 10% heat-inactivated fetal calf serum (FCS), 2 mM L-glutamine and antibiotic/antimycotic (Invitrogen, Grand Island, NY) at 37°C and 5% CO_2_ in a humidified atmosphere. Cells used were mycoplasma-negative as determined by direct and indirect testing in the Cleveland Clinic Cell Culture Core.

### *In vivo* tumor studies

Animals (B16F10 tumor experiments: 2-3 month old male & female (*Scarb1*); 3-4 month old female (WT, A1^−M/−M^, G1^−M/−M^ and DKO) and MB49 bladder cancer experiments: 6-7 month old males (WT, A1^−M/−M^, and DKO)) were inoculated subcutaneously on both flanks with 10^5^ tumor cells (in DMEM) per site. Tumor volume, based on caliper measurements, was calculated 3 times a week according to the ellipsoid volume formula: tumor volume = (the shortest diameter)^2^ x the largest diameter x 0.525. Tumor volume is expressed as mean value where n= number of tumor inoculation sites (two per animal).

### Flow cytometry

Splenocytes, were isolated by mechanical disruption. Individual spleens were cut into small pieces with sterile razor blade in cold RPMI medium and gently pressed through a 70 μm cell strainer using the rubber end of a syringe plunger. Cells were collected by centrifugation (300 x g, 7 min at 4°C) before being lysed for RBCs (LCK lysis buffer; A10492, Invitrogen). Individual tumors were chopped and digested using a tumor dissociation kit (130-096-730, Miltenyi Biotec Inc, San Diego, CA, USA) and the mouse impTumor-02 program of the gentleMACS dissociator (Miltenyi Biotec Inc, San Diego, CA, USA) according to manufacturer's protocol. Cells were lysed for RBC before surface staining. Fluorochrome-conjugated antibodies used were as follows: CD11b (CD11b-PE, clone M1/70, 12-0112, CD11b-FITC, clone M1/70, 11-0112, eBioscience), GR1 (GR1-AF700, clone RB6-8C5, 557979, BD Pharmingen), Ly-6C (Ly-6C-APC, clone AL-21, 560595, BD Pharmingen), Ly-6G (Ly-6G-PE, clone 1A8, 551461, BD Pharmingen), F4/80 (F4/80-FITC, clone BM8, 11-4801, eBioscience), CD45 (CD45 VioGreen; clone 30F11.1, 130-097-294, Miltenyi Biotec), CD3 (CD3-AF700, clone 17A2, 561388, BD Pharmingen), CD4 (CD4-FITC, clone RM4-5, 11-0042, eBioscience), CD8a (CD8a-PerCP, clone 53-6.7, BD Pharmingen). Cells were gated on Live (Live/Dead Violet, L34955, Invitrogen), were acquired on a BD LSR II FACS machine and data analyzed using FlowJo software (Treestar Inc., Ashland, OR).

### Lipid and complete blood count analyses

Blood was obtained by cardiac puncture at the time of sacrifice. Whole blood was run undiluted on an Advia 120 hematology system (Siemens Medical Solutions Malvern, PA, USA) for complete blood counts and differentials. For total cholesterol, and HDLc levels, EDTA-plasma was prepared and analyzed on a Roche c311 clinical autoanalyzer in a CAP and CLIA accredited reference laboratory in the Center for Cardiovascular Diagnostics and Prevention at the Cleveland Clinic.

### Statistical analysis

For tumor growth, error bars represent standard error of the mean (SEM). For CBC (Table 1) values are expressed as mean ± SEM. One way ANOVA (Analysis of variance) was used to analyze data across three or four genetic groups. Statistical differences between two groups were assessed by Student's *t* –test whereby a probability value of *p*<0.05 was considered significant.
